# Anti-Müllerian hormone and inhibin B dynamics in polycystic ovary syndrome: correlation with controlled ovarian hyperstimulation outcomes and pregnancy success

**DOI:** 10.3389/fendo.2025.1627560

**Published:** 2025-09-19

**Authors:** Yueying Li, Lu Han, Ying He, Xiaoyan Li, Yan Zhang, Huiying Zhang, Yingmei Wang, Huijuan Zhang, Wenyan Tian

**Affiliations:** ^1^ Department of Gynecology and Obstetrics, Tianjin Medical University General Hospital, Tianjin, China; ^2^ Tianjin Key Laboratory of Female Reproductive Health and Eugenic, Tianjin Medical University General Hospital, Tianjin, China

**Keywords:** polycystic ovary syndrome, controlled ovarian hyperstimulation, anti-müllerian hormone, inhibin b, pregnancy

## Abstract

**Purpose:**

To investigate the dynamic changes of serum Anti-Müllerian Hormone (AMH) and Inhibin B (INHB) during controlled ovarian hyperstimulation (COH) in patients with polycystic ovarian syndrome (PCOS) and analyze their correlation with COH outcomes and pregnancy success.

**Methods:**

A total of 40 individuals diagnosed with PCOS and 40 control subjects were recruited for the study. Serum concentrations of AMH and INHB were quantified at several key time points: at the initiation of gonadotropin treatment (dGn), on the fifth day of stimulation (dGn5), on the day of hCG administration (dhCG), on the day of oocyte pick-up (dOPU), and within the follicular fluid (FF) collected on the day of oocyte retrieval, employing the ELISA method for analysis. The changes in their concentrations were explored, and their correlations with COH outcomes and pregnancy success were analyzed.

**Results:**

AMH peaks on the basal day and subsequently declines during the COH procedure. The serum AMH levels are consistently correlated with basel testosterone(T), antral follicle count(AFC), estradiol (E2) on the trigger day, retrieved oocytes, two pronuclei (2PN) fertilization, available embryos, top-quality embryos (TQE) and TQE rate (p<0.05). INHB elevates during the COH process, peaks at dHCG, and thereafter declines. Serum levels are closely associated with AFC, E2 on the trigger day, retrieved oocytes, 2PN fertilizations, available embryos and TQE rate(p<0.05). During COH, AMH and INHB in both FF and serum are interrelated. AMH levels on dOPU (β=-5.2250, P=0.0014) and INHB levels on dOPU (β=-0.1106, P<0.05) were significant negative predictors of the TQE rate. Serum INHB at dGn5 demonstrated predictive value for pregnancy outcomes (AUC = 0.71, 95% confidence interval: 0.59–0.83).

**Conclusion:**

During COH, serum AMH and INHB exhibit cyclical variations. Serum AMH and INHB especially on dOPU are closely associated with the outcomes of COH. They possess the capacity to predict the results of COH. Moreover, serum INHB (dGn5) may serve as a potential biomarker for individualized prediction of pregnancy success in PCOS patients.

## Introduction

Globally, approximately 11% to 13% of female patients suffer from Polycystic Ovary Syndrome (PCOS), which poses a huge burden on the world economy and the medical field (1, 2). PCOS, a complex and commonly occurring endocrine disorder, represents a substantial threaten to women’s reproductive health. Its core symptoms, including irregular menstruation, polycystic ovarian morphology, and hyperandrogenemia, collectively impact the female reproductive system, leading to abnormalities in follicular development and ovulation processes, thereby compromising patients’ fertility (3). Consequently, PCOS is not only a reproductive issue but a multifaceted health concern involving endocrine and metabolic aspects.

PCOS is often associated with infertility risks (4, 5). *In Vitro* Fertilization and Embryo Transfer (IVF-ET) through Assisted Reproductive Technology (ART) offers potential solutions for many PCOS patients. However, the abnormal reproductive physiological mechanisms in PCOS patients, such as follicular development abnormalities and hormonal disturbances, adversely affect the efficacy and success rates of IVF-ET (6).

Recent advancements in PCOS research have identified numerous biomarkers that are critical in understanding its pathogenesis and treatment outcomes. Anti-Müllerian Hormone (AMH) and Inhibin B (INHB) are especially significant, exhibiting different expression levels in individuals with PCOS, underscoring their possible involvement in the progression of PCOS (7, 8). AMH, a member of the Transforming Growth Factor-β (TGF-β) Superfamily, serves as a marker for ovarian reserve and shows a correlation with antral follicle counts. In individuals with PCOS, elevated AMH levels can be linked to a greater number of pre-antral and antral follicles. This phenomenon may arise from both the accumulation of these follicles and an enhanced production by granulosa cells. This hormonal increase potentially hinders folliculogenesis, contributing to the anovulation typically observed in PCOS (9). It is also indicative of ovarian responsiveness during COH (10–12). Certain research indicate that AMH exhibits a declining tendency during the COH procedure (13–15). This change indicates the gradual development of follicles into mature ones during the ovulation induction process (14). INHB, another member of the TGF-β superfamily, is mainly secreted by granulosa cells in females. PCOS patients exhibit high INHB expression. Elevated INHB levels promote the degradation of gonadotropins within follicular cells, leading to follicular developmental disorders and antral follicle recruitment (16). Studies have shown that INHB exhibits a progressive increase during COH procedure, followed by a decline post-trigger day (17). Currently, there is insufficient evidence regarding the variations of INHB during the COH procedure in patients with PCOS. During COH, the dynamic changes of AMH and INHB, may reflect ovarian function and follicular development status, thereby influencing the outcomes of COH and pregnancy success. However, current research on dynamic changes and predictive value of these two hormones during COH remains relatively scarce.

Therefore, this study aims to systematically observe and analyze the dynamic variations in AMH and INHB levels during COH in PCOS patients and further explore the correlation between these changes and COH outcomes. This will not only contribute to a more comprehensive insight of the reproductive physiological mechanisms in PCOS patients but also provide important scientific evidence and potential therapeutic targets for predicting COH outcomes and pregnancy success, optimizing IVF-ET treatment protocols, and enhancing treatment success rates. Through this study, we hope to offer more precise and effective treatment strategies for PCOS patients, helping them achieve their fertility dreams.

## Materials and methods

### Study population and inclusion criteria

Patients having *in vitro* fertilization and embryo transfer at the General Hospital of Tianjin Medical University between October 2023 and March 2025 were enrolled in the study. For this study, researchers used the Rotterdam criteria to classify participants into two groups: those with normal ovarian function and those with PCOS. The gonadotropin-releasing hormone antagonist (GnRH-A) protocol was used to induce ovulation in both groups. Patients underwent either fresh embryo transfer or frozen-thawed embryo transfer (FET), with FET protocols including natural cycles, artificial cycles, and stimulated cycles.

Inclusion Criteria for the PCOS Group were based on meeting two of these three factors, after ruling out other causes of elevated androgen levels and ovulatory disorders (1): irregular or absent ovulation (2), clinical or biochemical signs of elevated androgens (3), polycystic ovarian morphology on ultrasound.

#### Control group

Female patients of normal reproductive age undergoing IVF treatment due to tubal factors or male factors during the same period as the PCOS group were selected. They had normal baseline endocrine test results, regular menstrual cycles, and no polycystic or abnormal changes in bilateral ovaries and no abnormal uterine sonographic findings on B-mode ultrasound.

#### Exclusion criteria

Both groups excluded patients with thyroid diseases, adrenal diseases, those who had not taken hormonal medications for at least 3 months, those with a family history of chronic hypertension or diabetes, those with other medical conditions and complications, those with endometriosis, and those with organic lesions in the reproductive system on transvaginal B-mode ultrasound.

### Collection of serum and follicular fluid

**Figure 1:**
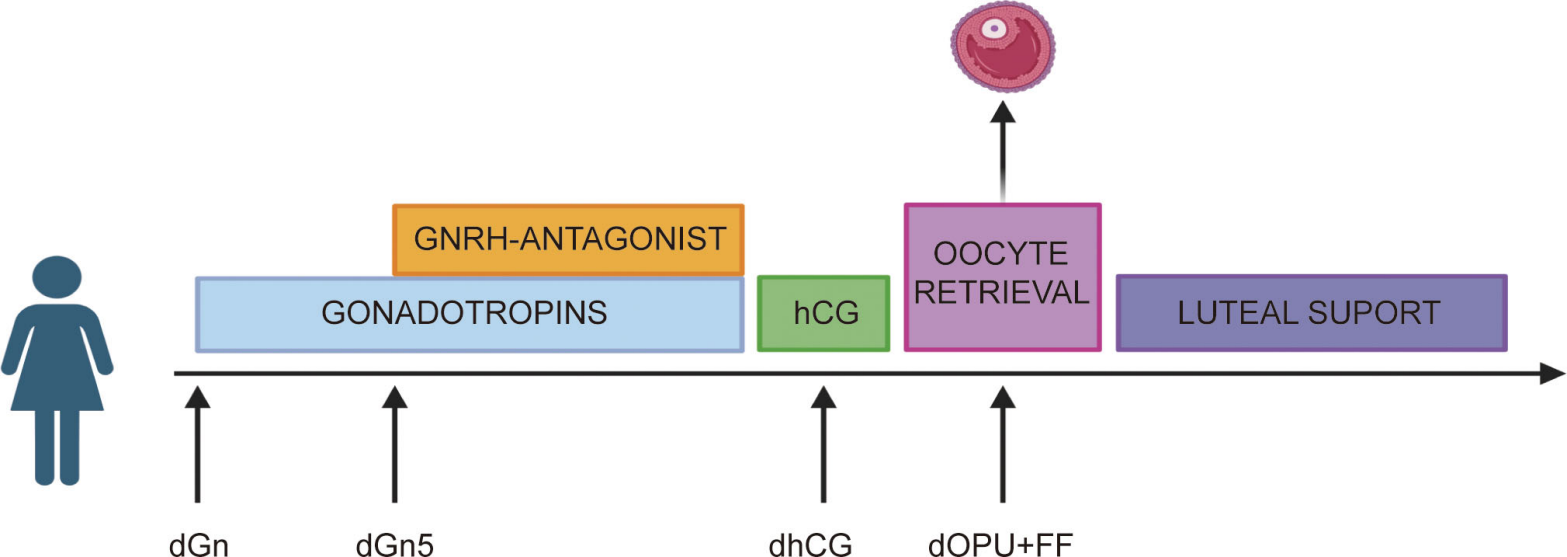
The plan for taking blood and FF. For all the study patients, Elbow venous blood samples (5 mL) were collected on dGn, dGn5, dhCG and dOPU. Concurrently, on the day of oocyte pickup, FF was also collected.

Both groups underwent the same ovulation induction protocol, namely GnRH-A protocol. From the second to the third day of menstruation, gonadotropin (Gn) was administered at a dosage of 150–225 IU daily. Ganirelix acetate (0.25 mg/day) was administered subcutaneously starting when the leading follicle achieved ≥14 mm diameter, and continued daily until the day of ovulation trigger (hCG administration). A dosage of 5000–10000 IU of hCG or 250 µg of recombinant hCG was administered after two or three dominant follicles had grown to 18 mm in diameter, and the levels of estradiol per mature follicle varied from 200 to 300 ng/L. A transvaginal aspiration was used to retrieve oocytes 36 to 38 hours following hCG treatment, with the use of ultrasound guidance. On certain days—the day of hCG injection (dhCG), the day of oocyte pickup (dOPU), the day of starting Gn treatment (dGn), and the fifth day of Gn stimulation (dGn5) —blood samples (5 mL) were taken. On the day of oocyte retrieval, follicles measuring 18 mm or larger were harvested for FF (**Figure 1**).

Prior to further analysis, all samples were spun at 3000 rpm for 10 minutes. The resulting supernatant was then kept at -80 °C in 1.5 mL EP tubes. In order to quantify AMH and INHB in serum and FF, ELISA kits were utilized.

### Enzyme-linked immunosorbent assay

The samples were retrieved from the -80 °C freezer and thawed in a 4 °C refrigerator until completely melted before proceeding with the assay. Prior to use, all reagents from the ELISA kits were allowed to reach room temperature (18-25 °C). The ELISA kits were employed to detect human INHB (Cat. No. E-EL-H0313, Elabscience) and human AMH (Cat. No. E-OSEL-H0004, Elabscience).

### Data collection for PCOS diagnostic markers and COH outcomes

Clinical data were retrospectively extracted from the Electronic Medical Record system for patients diagnosed with PCOS undergoing the IVF/ICSI cycle. Key indicators included: PCOS diagnostic markers (Basal testosterone (T, ng/ml) measured on cycle days 2-4; Antral Follicle Count (AFC) assessed via transvaginal ultrasound on days 2-4); COH treatment parameters (Total gonadotropins dosage (IU); Duration of COH (days); Serum E2 level on trigger day (pg/ml)); and Embryology laboratory outcomes (Number of retrieved oocytes; 2PN fertilizations; Available embryos; Top-quality Embryo (TQE)). TQE was specifically defined according to the Istanbul Consensus as top-quality cleavage-stage embryos on Day 3: possessing 8 cells (or 7–9 cells), ≤10% fragmentation, uniform blastomere size, and absence of multinucleation (18). TQE rate (%) was calculated accordingly. Data extraction was performed by two independent clinicians using a dual-blinded approach with cross-verification for accuracy.

### Statistical analysis

Statistical analysis was performed using GraphPad Prism 9.0 software. During the data preprocessing phase, all variables were initially subjected to normality testing using the Shapiro-Wilk test. Normally distributed quantitative data were expressed as mean ± standard deviation (Mean ± SD), with intergroup comparisons conducted using independent samples t-test (for two groups) or one-way ANOVA (for multiple groups) according to sample size characteristics. Conversely, non-normally distributed quantitative data were presented as median (interquartile range) [M(QR)], with between-group differences analyzed through Mann-Whitney U test (for two groups) or Kruskal-Wallis H test (for multiple groups). Categorical variables were described using frequency (percentage). The linear correlation between variables was assessed using Pearson correlation coefficient, while the association analysis of binary variables was performed through receiver operating characteristic (ROC) curve analysis. For all statistical tests, a two-tailed P-value <0.05 was considered the threshold for determining statistical significance.

### Ethics

The research was approved by the Ethics Committee of Tianjin Medical University General Hospital before it was conducted. After being apprised of their rights, possible advantages, and hazards, all participants were asked to sign informed consent forms (Ethics: IRB2023-KY-332).

## Results

### Comparison of general characteristics and COH outcomes of patients

Forty patients with PCOS and forty controls were part of an 80-person IVF cohort (**Table 1**). There were substantial differences in the baseline levels of testosterone and luteinizing hormone, with the former having significantly higher levels (P<0.05, P<0.01). Furthermore, there was a significant increase in the number of antral follicles in the group with PCOS (P<0.0001). Among the other baseline variables, there were no significant differences between the groups (P>0.05). Remarkably, there were differences in the PCOS group with regard to the number of days of gonadotropin administration needed, E2 levels on the trigger day, number of oocytes recovered, two pronuclei (2PN) fertilization and available embryos (P<0.05, P<0.0001, P<0.0001, P<0.0001, P<0.001). Surprisingly, the PCOS group had a lower rate of top-quality embryos (TQE) (P<0.0001).

**Table 1:** Comparison of general characteristics and COH outcomes of patients.

Item	PCOS group	Control group	*P*
Age, years	32.08 ± 4.15	32.72 ± 3.35	0.53
BMI (kg/m2)	23.73 ± 4.48	22.87 ± 3.07	0.41
Previous pregnancies	0.40 ± 0.71	0.69 ± 1.26	0.31
AFC	25.08 ± 4.88	14.69 ± 4.46	<0.0001
Basal E2(pg/ml)	29.48 ± 10.72	31.40 ± 13.67	0.57
Basal P(ng/ml)	0.30 ± 0.31	0.31 ± 0.12	0.92
Basal T(ng/ml)	38.85 ± 15.21	30.88 ± 9.43	0.025
Basal FSH(mIU/ml)	6.15 ± 1.47	6.56 ± 1.53	0.32
Basal LH(mIU/ml)	4.59 ± 2.30	3.30 ± 1.06	0.0089
Basal PRL(ng/ml)	17.07 ± 8.76	18.97 ± 8.76	0.44
Gonadotropins dosage (IU)	2361.29 ± 868.67	2351.97 ± 695.90	0.9616
Duration of COH(d)	10.61 ± 1.995	9.71 ± 1.55	0.0436
E2 on the trigger day(pg/ml)	4576.93 ± 2533.34	2334.27 ± 970.79	<0.0001
Retrieved oocytes	26.00 ± 14.0.31	13.81 ± 5.17	<0.0001
2PN fertilizations	15.68 ± 8.43	8.61 ± 3.51	<0.0001
Available embryos	15.19 ± 8.45	8.53 ± 3.53	0.0002
TQE	6.19 ± 4.84	4.53 ± 2.87	0.0999
TQE rate (%)	22.38 ± 14.42	49.99 ± 22.85	<0.0001

BMI, Body Mass Index; AFC, Antral Follicle Count; E2, Estradiol; P, Progesterone; T, Testosterone; FSH, follicle stimulating hormone; LH, luteinizing hormone; PRL, Prolactin; COH, Controlled Ovarian Hyperstimulation; 2PN, two pronuclei; TQE, Top-Quality Embryo.

### Levels of AMH and INHB in serum and FF during COH

Research indicates that serum AMH concentrations in the PCOS cohort were elevated throughout the COH, showing a marked statistical difference (P<0.0001). Notably, serum AMH levels displayed a declining trend in both groups. When comparing the serum AMH levels of dGn5, dhCG, and dOPU with baseline levels within each group, it was observed that in the PCOS cohort, levels at dhCG and dOPU were reduced compared to dGn, with significant differences noted (P<0.0001, P<0.0001). A similar pattern was observed in the control group (P<0.001, P<0.0001). However, on days dGn5, dhCG, and dOPU, INHB levels in the PCOS group, reaching significance (P<0.0001, P = 0.0001, P<0.01). The trajectory of serum INHB levels increased from dGn to dhCG and subsequently decreased at dOPU. Comparative analyses within each group from baseline levels on dGn5, dhCG, and dOPU revealed that INHB levels in the PCOS group were higher on dGn5, dhCG and dOPU compared to dGn, with significant differences (P<0.01, P<0.0001, P<0.01). In the control group, INHB levels on dhCG and dOPU exceeded those on dGn, marking significant differences (P <0.0001, P<0.001). On the day of oocyte pickup, FF AMH levels were significantly higher in the PCOS group (P=0.0002). This was also true for INHB levels, which were elevated in the PCOS group (P<0.05) (**Table 2**).

**Table 2:** Levels of AMH and INHB in serum and FF during COH.

Item	AMH (ng/ml)			INHB (pg/ml)		
	PCOS group	Control group	*P*	PCOS group	Control group	*P*
dGn	8.22 ± 3.48	2.22 ± 1.10	<0.0001	10.45 ± 6.21	10.32 ± 6.09	0.9384
dGn5	6.48 ± 3.06	1.96 ± 1.21	<0.0001	75.27 ± 63.35^**^	25.34 ± 16.20	<0.0001
dhCG	4.27 ± 2.18^****^	1.27 ± 0.79^***^	<0.0001	165.21 ± 107.43^****^	77.07 ± 54.56^****^	0.0001
dOPU	3.48 ± 1.63^****^	1.05 ± 0.70^****^	<0.0001	81.72 ± 65.42^**^	44.73 ± 40.80^***^	0.0072
FF	9.62 ± 8.63	3.10 ± 1.92	0.0002	234386.99 ± 96184.71	164787.57 ± 66059.82	0.0112

Asterisks represent comparison of AMH/INHB levels at each time point vs. baseline (dGn). *P<0.05; **P<0.01; ***P<0.001; ****P<0.0001.

### Correlation between the levels of AMH and INHB in serum and FF at different time points

**Figure 2:**
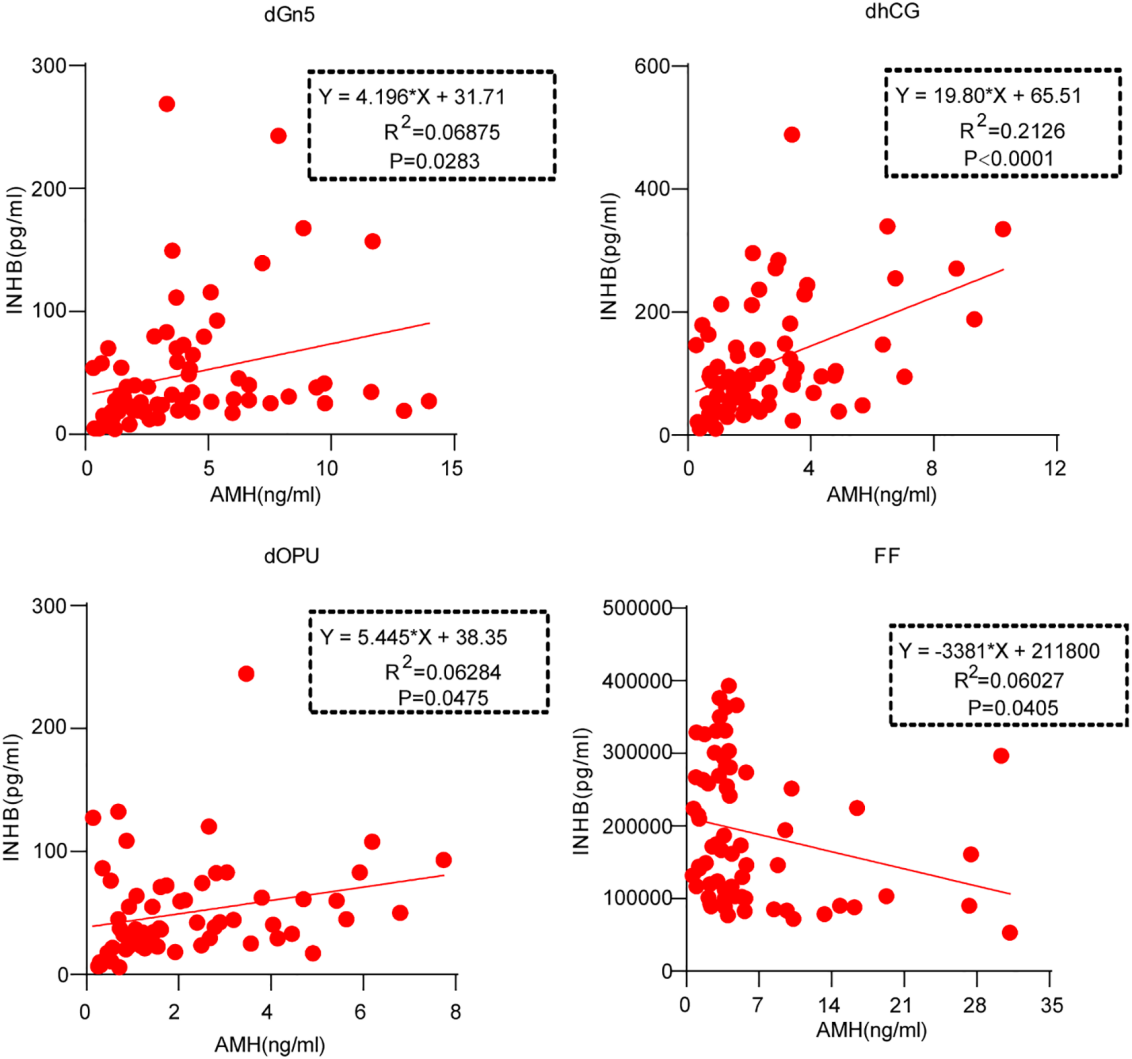
Correlation between the Levels of AMH and INHB in Serum and FF at the different time points in patients.

Figure analysis illustrates correlations between AMH and INHB levels at several time points (dGn5, dhCG, dOPU) and in FF. On dGn5, a positive correlation is noted between AMH and INHB levels (P<0.05), suggesting that increases in AMH levels are generally accompanied by rises in INHB levels, though this association is not particularly robust (R^2^ = 0.0688). At dhCG, the correlation strength between AMH and INHB levels intensifies (P<0.0001), with data demonstrating a more pronounced upward trajectory, indicating that rises in AMH are likely paralleled by significant increases in INHB levels (R^2^ = 0.2126). At dOPU, the relationship persists (P<0.05), with the strength of correlation remaining moderate (R^2^ = 0.0628). In FF, a slight negative correlation emerges between AMH and INHB levels (P < 0.05), where increases in AMH levels correspond with minor decreases in INHB levels (R^2^ = 0.0603). Overall, the correlation strength varies across different time points and in FF, with dhCG demonstrating the strongest positive correlation (**Figure 2**).

### Correlations of serum and FF AMH levels with PCOS diagnostic markers and COH outcomes

This study employed Pearson linear correlation analysis to evaluate the associations between serum and follicular fluid AMH levels and PCOS diagnostic markers and COH outcomes. Serum AMH levels at dGn, dGn5, dhCG, and dOPU showed positive correlations with AFC, trigger-day E2 levels, number of oocytes retrieved, number of 2 pronuclei (2PN) embryos, and number of usable embryos, while demonstrating negative correlations with TQE rate. Additionally, AMH levels at dGn5, dhCG, and dOPU showed positive correlations with baseline T. However, AMH at dGn showed no significant correlation with baseline T. Moreover, serum AMH levels at dGn, dhCG, and dOPU were positively correlated with the number of TQE. No statistically significant correlations were observed between serum AMH levels and Gn dosage or duration of COH. Follicular fluid AMH levels exhibited a positive correlation with AFC, while demonstrating a negative correlation with the TQE rate, however, no significant associations were observed with Gn dosage, COH duration, trigger-day E_2_ levels, number of oocytes retrieved, 2PN parameters, number of usable embryos, or number of TQE. (detailed correlation coefficients and p-values are presented in **Table 3**).

**Table 3:** Correlations of serum and FF AMH levels with PCOS diagnostic markers and COH outcomes.

Item	dGn	dGn5	dhCG	dOPU	FF
Basal T(ng/ml)	0.2045	0.2600^*^	0.2606^*^	0.2487^*^	0.0946
AFC	0.6583^****^	0.6339^****^	0.6160^****^	0.6520^****^	0.4110^***^
Gonadotropins dosage (IU)	-0.0504	-0.0311	-0.0172	-0.0044	-0.1404
Duration of COH(d)	0.1461	0.1206	0.0828	0.1556	0.0036
E2 on the trigger day(pg/ml)	0.6713^****^	0.5036^****^	0.5089^****^	0.5157^****^	0.1570
Retrieved oocytes	0.6978^****^	0.5050^****^	0.4674^****^	0.4641^****^	0.1189
2PN fertilizations	0.6648^****^	0.4443^***^	0.4549^***^	0.4588^****^	0.1621
Available embryos	0.6583^****^	0.4261^***^	0.4306^***^	0.4378^***^	0.1227
TQE	0.3215^*^	0.2092	0.2625^*^	0.2662^*^	-0.0083
TQE rate (%)	-0.4179^**^	-0.3954^***^	-0.3709^**^	-0.3604^**^	-0.3084^**^

*P<0.05; **P<0.01; ***P<0.001; ****P<0.0001.

T, Testosterone; AFC, Antral Follicle Count; COH, Controlled Ovarian Hyperstimulation; E2, Estradiol; 2PN, two pronuclei; TQE, Top-quality Embryo.

### Correlations of serum and FF INHB levels with PCOS diagnostic markers and COH outcomes

This study employed Pearson linear correlation analysis to evaluate the associations between serum and follicular fluid INHB levels and PCOS diagnostic markers and COH outcomes. On dGn5, serum INHB concentrations exhibited a positive correlation with AFC, E2 levels on the trigger day and the number of oocytes collected, while demonstrating a negative correlation with the TQE rate. At dhCG, INHB concentrations exhibited a positive correlation with AFC, E2 levels on the trigger day, the quantity of oocytes retrieved, the incidence of 2PN fertilizations, and the count of viable embryos. In the context of dOPU, there was a positive correlation observed between INHB concentrations and both COH duration and E2 levels on the trigger day. Conversely, a negative correlation was noted with the TQE rate. No significant correlations were found between serum INHB levels on dGn and those in FF, nor with Gn dosage, COH duration, E2 levels on the trigger day, the number of oocytes retrieved, the number of 2PN fertilizations, the count of viable embryos, or the TQE rate (detailed correlation coefficients and p-values are presented in **Table 4**).

**Table 4:** Correlations of serum and FF INHB levels with PCOS diagnostic markers and COH outcomes.

Item	dGn	dGn5	dhCG	dOPU	FF
Basal T(ng/ml)	0.0281	-0.0680	0.0640	-0.0599	0.0075
AFC	0.1618	0.2976^*^	0.3396^**^	0.2089	-0.0524
Gonadotropins dosage(IU)	-0.1218	0.0854	0.0528	0.1062	-0.0749
Duration of COH(d)	-0.1616	0.0912	0.1550	0.2860^*^	-0.0766
E2 on the trigger day(pg/ml)	-0.0658	0.3638^**^	0.5370^***^	0.4228^**^	0.0602
Retrieved oocytes	-0.1580	0.2958^*^	0.3989^**^	0.2211	-0.0030
2PN fertilizations	-0.1709	0.2309	0.2580^*^	0.1110	0.0299
Available embryos	-0.1677	0.2398	0.2642^*^	0.1248	0.0553
TQE	-0.1928	0.1795	0.1446	-0.0355	0.0447
TQE rate (%)	-0.0801	-0.4087^**^	-0.1562	-0.3125^**^	0.0656

*P<0.05; **P<0.01; ***P<0.001.

T, Testosterone; AFC, Antral Follicle Count; COH, Controlled Ovarian Hyperstimulation; E2, Estradiol; 2PN, two pronuclei; TQE, Top-quality Embryo.

### Correlation between the levels of AMH and INHB on dOPU and the TQE rate

The outcome of the multiple linear regression analysis indicated that the regression model was significant. Specifically, after adjustment for age, BMI, and baseline LH, AMH levels on dOPU (β= -5.2250, P = 0.0014) and INHB levels on dOPU (β=-0.1106, P = 0.0252) remained significant negative predictors of the TQE rate. The adjusted model explained 22.6% of the variance in the rate of TQE. These results demonstrate that the dual-hormone prediction model incorporating both AMH and INHB exhibits superior predictive performance compared to single-hormone models (**Table 5**).

**Table 5:** Correlation between the levels of AMH and INHB on dOPU and the TQE rate.

Item	Unadjusted			Adjusted		
	β	95% Cl	*P*	β	95% Cl	*P*
dOPU AMH	-4.3610	-7.3720- -1.3561	0.0052	-5.2250	-8.3620- 2.0890	0.0014
dOPU INHB	-0.1094	-0.2015- -0.0174	0.0206	-0.1106	-0.2071- -0.0141	0.0252

Adjusted for age, BMI, and baseline LH.

### Comparison of cumulative pregnancy rates between the two groups

This study continued to track the pregnancy outcomes of patients in both groups and collected data to compare differences in cumulative pregnancy rates. Cumulative pregnancy rate is the probability of achieving at least one clinical pregnancy after transferring all available embryos (fresh + frozen) derived from a single oocyte retrieval cycle. The cumulative pregnancy rate was 47.50% (19/40) in the PCOS group and 77.50% (31/40) in the control group. Chi-square test results indicated a statistically significant difference in cumulative pregnancy rates between the two groups (χ²=7.68, P = 0.0056), with the PCOS group demonstrating a significantly lower cumulative pregnancy rate (47.50%) compared to the control group (77.50%) (**Table 6**).

**Table 6:** Comparison of cumulative pregnancy rates between the two patient groups.

Groups	Samples	Outcomes		χ² test	
		Pregnancy	Not pregnancy	χ²	*P*
PCOS	40	47.50% (19/40)	52.50% (21/40)	7.68	0.0056
Control	40	77.50% (31/40)	22.50% (9/40)		
Sum	80	50	30		

### Comparison of live birth rates between the two groups

Building upon these findings, the study continued to track pregnancy outcomes until live birth. As of March 2025, the PCOS group had undergone 71 embryo transfer cycles, resulting in 7 live births. The control group had 66 embryo transfer cycles with 18 live births. The live birth rate was calculated using the formula: live birth rate = (number of live birth cycles/number of embryo transfer cycles)×100. This yielded a live birth rate of 9.86% (7/71) in the PCOS group and 27.27% (18/66) in the control group. Chi-square analysis revealed a statistically significant difference in live birth rates between the two groups (χ²=6.95, P = 0.0084), with the PCOS group demonstrating a significantly lower live birth rate (9.86%) compared to the control group (27.27%) (**Table 7**).

**Table 7:** Comparison of live birth rates between the two patient groups.

Groups	Times	Outcomes		χ² test	
		Live birth	Not live birth	χ²	*P*
PCOS	71	9.86% (7/71)	90.14% (64/71)	6.95	0.0084
Control	66	27.27% (18/66)	72.73% (48/66)		
Sum	137	25	112		

### Association of AMH and INHB with pregnancy success

**Figure 3:**
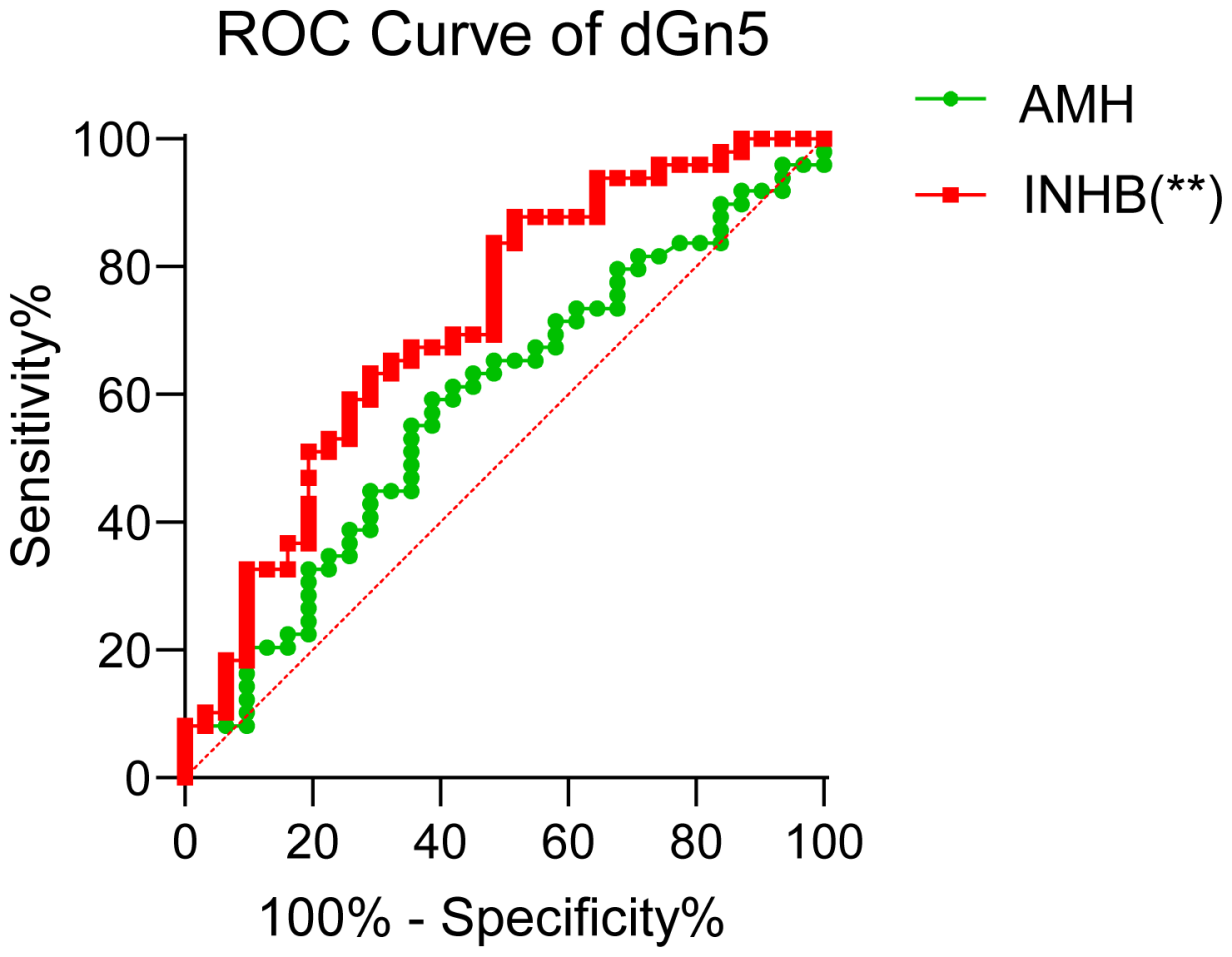
INHB predict pregnancy success in both groups of patients. **p < 0.01.

Following the completion of pregnancy outcome data collection in the two study groups, this research further explored the association between AMH, INHB, and clinical pregnancy outcomes. To investigate this association, we performed ROC curve analysis to evaluate the predictive capacity of these hormones for pregnancy outcomes. The ROC analysis revealed significant differences in the predictive efficacy of serum hormone levels and follicular fluid biomarkers at different timepoints. Specifically, serum INHB at dGn5 (day 5 of gonadotropin stimulation) demonstrated predictive value for pregnancy outcomes (AUC = 0.71, 95% confidence interval: 0.59–0.83). The optimal cutoff value for serum INHB at dGn5 was determined to be 29.15 pg/mL, with a sensitivity of 0.63 and specificity of 0.71. In contrast, neither serum AMH at any timepoint nor follicular fluid AMH and INHB levels showed significant predictive power, as their ROC curves did not exceed random prediction thresholds (AUC<0.65, P>0.05) (**Figure 3**). These findings suggest that serum INHB (dGn5) may serve as a potential biomarker for individualized prediction of pregnancy outcomes in PCOS patients.

## Discussion

This is a prospective study that involved the dynamic monitoring of AMH and INHB. This monitoring aims to forecast the results of COH and alleviate the reproductive challenges faced by patients with PCOS. Over findings align with the current body of research. In individuals diagnosed with PCOS, there was an observed increase in both serum and FF concentrations of AMH, which can be ascribed to a greater number of preantral and antral follicles (19). Furthermore, a heightened ratio of luteinizing hormone to follicle-stimulating hormone (LH/FSH) in patients with PCOS results in hormonal dysregulation and atypical aromatase expression, subsequently elevating androgen concentrations and enhancing AMH secretion from granulosa cells (GCs) (20). During the progression of COH in this study, a progressive decline in AMH levels was observed in both the PCOS and control cohorts. This trend is associated with the development of follicles and the decrease in the quantity of antral and preantral follicles (14). The relationship between follicular maturation during controlled ovarian hyperstimulation and the increasing concentrations of estradiol (E2) is well-established, as this hormone inhibits the production of AMH in GCs (21). Furthermore, following oocyte retrieval, certain granulosa cells are removed while others play a role in the development of the corpus luteum, thereby impacting the reduction in AMH levels. The univariate correlation analysis revealed a positive correlation between serum AMH concentrations measured at four distinct time points and E2 levels on the trigger day, the number of retrieved oocytes, the count of 2PN fertilizations, the number of viable embryos, and TQE. Nonetheless, the relationship observed between AMH levels across various stages (dGn, dGn5, dhCG, dOPU) and the TQE rate was inversely correlated, suggesting that AMH may serve as a predictive biomarker for the outcomes of ovarian hyperstimulation, thereby offering valuable clinical insights (22, 23).

INHB, identified in several studies as a predictor of COH outcomes, showed differing levels between the PCOS and control groups in past research. Nevertheless, this study did not observe significant differences in INHB on the baseline day, primarily because the baseline day coincided with the day of Gonadotropin (Gn) injection. Since INHB predominantly originates from developing preantral or antral follicles, which had not yet begun developing on the day of Gn administration, no significant disparities were noted between the groups (24). This may also pertain to the absence of notable differences in BMI among the chosen patient cohorts, given that INHB secretion is recognized to have an inverse relationship with BMI in both individuals with PCOS and those who are healthy (25, 26). In this experiment, the concentrations of INHB in the PCOS cohort exceeded those observed in the control group at later time intervals (dGn5, dhCG, dOPU), presumably attributable to the increased quantity of preantral and antral follicles present in patients with PCOS (27). Prior to hCG administration, an ascending trend in INHB levels was observed; however, following hCG administration, a decline occurred. This is likely due to the initial increase in Follicle-Stimulating Hormone (FSH) release post-Gn administration, which boosts INHB production through its feedback mechanism (28). After hCG administration, the cessation of Gn leads to a decrease in FSH secretion, reducing the feedback effect (29). Furthermore, during the advanced phase of follicular development, elevated levels of estrogen and progesterone suppress FSH secretion, resulting in decreased INHB levels (30). Elevated LH drives theca-cell androgen overproduction, which suppresses granulosa cell function via androgen receptor-mediated signaling. This directly inhibits INHB transcription and protein secretion. Particularly in PCOS patients, chronically high LH levels create a hyperandrogenic microenvironment that dysregulates steroidogenesis, further compromising granulosa cell capacity to produce INHB during late COH (31).The univariate analysis indicated a positive correlation between INHB levels on dGn5 and E2 levels on the trigger day, as well as the number of retrieved oocytes, while revealing a negative correlation with the TQE rate. The levels of INHB on dhCG exhibited a positive correlation with E2 levels on the trigger day, the quantity of retrieved oocytes, the occurrences of 2PN fertilizations, and the number of available embryos. INHB levels on dOPU exhibited a positive correlation with COH duration and E2 levels on the trigger day, while demonstrating a negative correlation with the TQE rate, highlighting the predictive significance of INHB for COH outcomes (32).

Both AMH and INHB are closely associated with the outcomes of COH. There might potentially be an inherent relationship between them as predictive indicators. In this study, through conducting a correlation analysis of the serum and FF at various time points, it was discovered that during the entire COH, AMH and INHB in the serum exhibited a positive correlation at time points other than the baseline day. This could be attributed to the fact that both hormones are derived from GCs and belong to the same hormonal family (14, 33). In the FF, a negative correlation was observed between AMH and INHB. This might be related to the distinct functions that AMH and INHB play during the normal ovulatory cycle, and is also closely associated with the mutual interactions among AMH, INHB, and FSH (34, 35).

In addition to the aforementioned findings, our results incorporated serum samples from dOPU, which was an addition compared to previous studies (14). Upon conducting separate univariate analyses, it was revealed that AMH and INHB in the serum at the newly added time point of dOPU were closely correlated with the rate of TQE in COH. This implies that the AMH and INHB on the day of dOPU are excellent predictors for COH outcomes. Consequently, a multiple linear equation was formulated, which further demonstrated the close association of AMH and INHB with COH outcomes. Furthermore, in comparison with the univariate analysis, the multivariate equation remarkably enhanced the predictive efficacy of the model. This discovery might be attributed to the fact that the blood samples on the day of dOPU are most similar to the physiological state in the FF.

The clinical characteristics of reproductive outcomes in PCOS patients have garnered significant attention. This study confirmed that PCOS patients exhibit significantly lower pregnancy rates and live birth rates compared to age-matched healthy controls. The reduced pregnancy rates in PCOS are closely associated with compromised embryo quality and impaired endometrial receptivity. Although the PCOS group demonstrated higher numbers of retrieved oocytes and 2PN during ovarian stimulation, their TQE rate was markedly decreased. This suggests that hyperandrogenism and insulin resistance may impair oocyte developmental competence through mechanisms involving oxidative stress, mitochondrial dysfunction, and aberrant epigenetic modifications (36). Furthermore, PCOS patients frequently exhibit dysregulated insulin signaling pathways and chronic low-grade inflammation in the endometrium, leading to diminished endometrial receptivity and reduced embryo implantation success (37, 38). Notably, even when ovulation is achieved via controlled ovarian hyperstimulation (COH), the live birth rate remains significantly lower in PCOS patients, highlighting the impact of extraovarian factors: Luteal phase insufficiency caused by hyperandrogenic environments impairs embryonic development. Elevated plasminogen activator inhibitor-1 (PAI-1) levels secondary to hyperinsulinemia are strongly associated with recurrent pregnancy loss (39). Serum INHB at dGn5 demonstrated predictive value for pregnancy outcomes, possibly reflecting critical oocyte-granulosa cell interactions during folliculogenesis. In contrast, AMH failed to show significant predictive utility in this cohort, consistent with prior reports (17).

This study has several limitations: First, the relatively small sample size may limit statistical power, particularly in subgroup analyses. Second, the short follow-up duration resulted in incomplete outcome data, as a proportion of patients remained clinically pregnant at the time of data cutoff. Furthermore, live birth outcomes are influenced by multifactorial determinants (e.g., pregnancy complications, infections), which precluded effective predictive modeling in this investigation.

## Conclusion

In summary, during ovulation induction, AMH and INHB in the PCOS and the control group exhibited cyclical changes. Specifically, AMH levels gradually decreased, while INHB levels first increased and then decreased. Serum AMH and INHB are closely related to COH outcomes and can predict them. Moreover, the levels of serum AMH and INHB on dOPU can jointly be utilized to forecast the rate of TQE. Serum INHB (dGn5) may serve as a potential biomarker for individualized prediction of pregnancy outcomes in PCOS patients.

## Data Availability

The original contributions presented in the study are included in the article/supplementary material. Further inquiries can be directed to the corresponding authors.
